# Successful treatment of a rare extended retroperitoneal necrotizing soft tissue infection caused by extended-spectrum beta-lactamase-producing *Escherichia coli*: A case report

**DOI:** 10.1097/MD.0000000000005576

**Published:** 2016-12-09

**Authors:** Rui He, Xin Qi, Bing Wen, XiangYan Li, Li Guo

**Affiliations:** a Department of Plastic Surgery and Burn; b Department of Anti-infection; c Department of Radiology, Peking University First Hospital, Beijing, People's Republic of China.

**Keywords:** ESBL-producing *Escherichia coli*, necrotizing soft tissue infection, retroperitoneal

## Abstract

**Rationale:**

Retroperitoneal necrotizing soft tissue infection (NSTI) is a rare but life-threatening disease. Here, we present a case of extended retroperitoneal NSTI caused by extended-spectrum beta-lactamase (ESBL)-producing Escherichia coli (E coli).

**Patient concerns:**

The patient complained of progressive redness, swelling, and right flank pain for 10 days, extending to the scrotum for 1 day.

**Diagnoses:**

He was admitted with an initial diagnosis of cellulitis.

**Interventions:**

Debridement was performed after the scrotum developed necrosis on day 2 of hospitalization. The source of infection was found to be an idiopathic retroperitoneal abscess, which was confirmed by computed tomography. Two consecutive microbiological cultures (aerobic plus anaerobic) of the tissue revealed the presence of ESBL-producing E coli. With the application of negative pressure wound therapy (NPWT), we sutured the wound after consecutive debridement.

**Outcomes:**

During the 32 months of follow-up, the patient recovered very well and felt extremely satisfied.

**Lessons:**

This case reminds us that ESBL-producing E coli can cause retroperitoneal abscesses, which may induce NSTI. Aggressive debridement and broad-spectrum antibiotics should be administrated immediately when NSTI is suspected, and NPWT is an effective adjuvant therapy for wound closure.

## 1 Introduction

Necrotizing soft tissue infections (NSTI) are infrequent but highly lethal infections, which can be defined as infection of any of the layers within the soft tissue compartment (dermis, subcutaneous tissue, superficial fascia, deep fascia, or muscle) that are associated with necrotizing changes.^[1]^ Typical sites of necrotizing fasciitis are the lower extremities, abdomen, and perineum.^[2,3]^ Here, we present a rare case of extended retroperitoneal NSTI caused by an idiopathic retroperitoneal abscess involving the right flank, lower abdomen, perineum, and scrotum.

## 2 Case report

A 50-year-old man was referred to the Department of Anti-infection in our hospital because of progressive redness, swelling, and right flank pain for 10 days, extending into the scrotum for 1 day. He had mild diarrhea and anorexia and was deeply depressed. He had a history of diabetes mellitus (DM) for 15 years without regular treatment and only recently took traditional Chinese medicine but has not monitored his blood sugar. He was admitted with an initial diagnosis of cellulitis. Approval was obtained from the hospital's ethics committee, and patient consent was obtained.

His blood pressure was 125/80 mm Hg, his heart rate was 92 beats/min, his respiratory rate was 18 breaths/min, and his body temperature was 37.9°C. His body mass index was 32.1. His abdomen was soft, but the flank and right lower abdomen displayed tenderness. Blood tests revealed the following: white blood cell count 21.4×10^8^/L, platelets 409 × 10^9^/L, hemoglobin 161 g/L, creatinine 64.9 umol/L, albumin 18.8 g/L, alanine aminotransferase 22 IU/L, prothrombin time 17.3 seconds (normal control 9.8–12.4 s), partial thromboplastin time 35.0 seconds (normal control 26.9–37.6 s), blood sugar 15.3 mmol/L, glycated hemoglobin 11.7%, erythrocyte sedimentation rate 76 mm/h, and C-reactive protein 272.32 mg/L. Ultrasound revealed that the subcutaneous soft tissue of the right abdominal wall was edematous.

**Figure 1:**
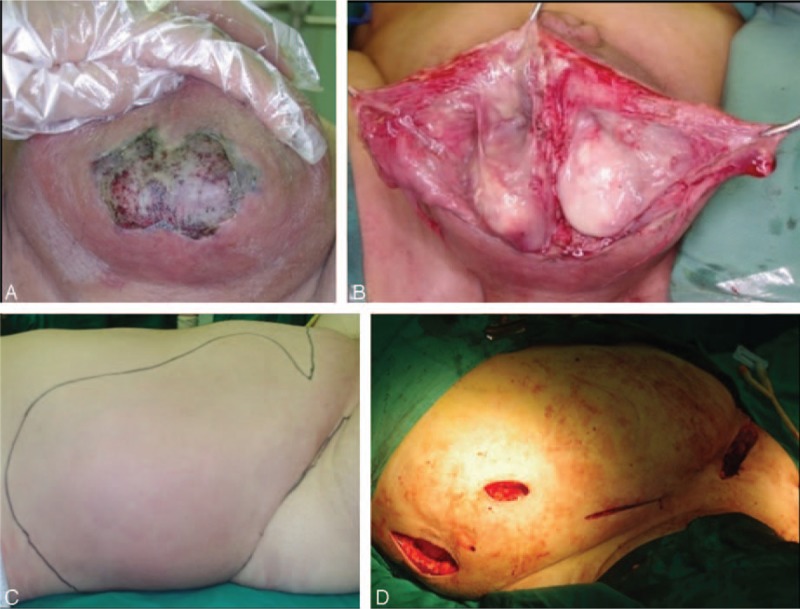
(A) The skin of scrotum was necrotized. (B) Severe inflammatory necrosis was found in the scrotum with abundant foul odor and brown fluid. (C) The extent of the inflamed skin was outlined. (D) Four additional incisions.

**Figure 2:**
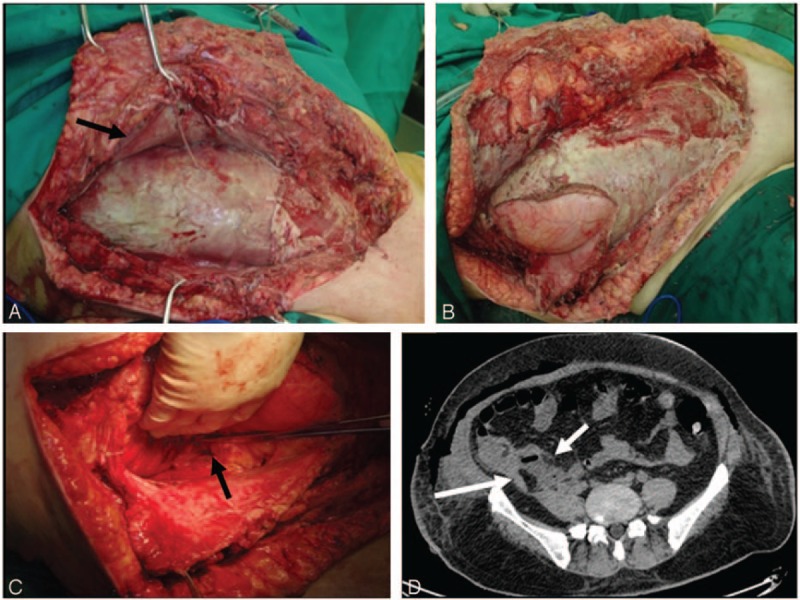
(A) The obliquus externus abdominis (arrow) was cut, and the aponeurosis of obliquus internus abdominis was infected. (B) The obliquus internus abdominis and transversus abdominis were cut. (C) A sinus tract (arrow) was traced which directly extended to the retroperitoneum. (D) Axial CT scan revealed a retroperitoneal abscess (short arrow), measuring 6.5 cm × 3.3 cm × 3.7 cm (vertical × anteroposterior × transverse), and the appendix (long arrow) was intact with mild swelling. CT = computed tomography.

**Figure 3:**
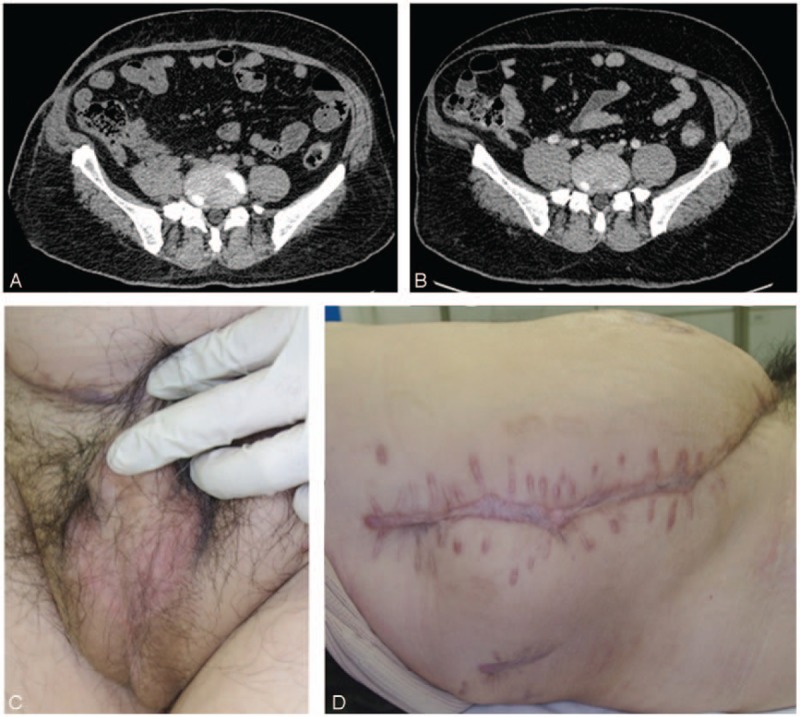
(A) Axial CT scan revealed that the abscess was partially absorbed on day 61, measuring 2.5 cm × 1.7 cm × 2.0 cm (vertical × anteroposterior × transverse). (B) Axial CT scan revealed that the abscess was completely absorbed 6 months later. (C) The appearance of the scrotum 6 months later. (D) The appearance of the flank 6 months later. CT = computed tomography.

The patient was administered intravenous antibiotics (amoxicillin sodium, clavulanate potassium 2.4 g q.8 h, and moxifloxacin hydrochloride 0.4 g daily) immediately, but his temperature rose to 38.7°C the first night. On day 2, the skin of the scrotum had developed necrosis and was markedly painful (Fig. 1A). An emergency operation was performed after our consultation based upon the suspicion of Fournier gangrene (FG). Severe inflammatory necrosis was found in the scrotum with abundant foul odor and brown fluid (Fig. 1B). Wide debridement was performed, and the infection was found to have spread to the suprapubic area along the Colle's fascia, but only small amounts of necrotic tissue were found in the suprapubic area. Three exploratory incisions were made over the inflamed lower abdomen and flank (Fig. 1C) to the depth of the Scarpa's fascia (Fig. 1D), but the tissues seemed to be normal. All the incisions were filled with iodophor gauze. We upgraded the antibiotics to norvancomycin (0.8 g q.12 h) and meropenem (1 g q.8 h) after surgery. Systemic supportive therapies, including plasma transfusion, albumin infusion, blood sugar control, and fluid and electrolyte balance, were administered simultaneously. On day 4, his body temperature dropped to normal and we arranged for the second debridement. Besides the necrosis of the scrotal dartos, a large quantity of purulent fluid was found in the deep layer of the suprapubic area. We then cut the obliquus externus abdominis and linked the 4 incisions together to have a complete view. Almost the entire aponeurosis of the obliquus internus abdominis was infected (Fig. 2A), purulent effusion was found again near the costal margin, and the obliquus internus abdominis and transversus abdominis were dissected layer by layer (Fig. 2B). The infection had spread to the posterior abdominal wall via the transverse fascia. The friable, loose fascia was easy to separate with finger dissection, and it was accompanied by a large area of fat necrosis. A sinus tract was traced that directly extended to the retroperitoneum for about 10 cm (Fig. 2C). We highly suspected that the end of this sinus tract would be the source of the infection. The wound was again covered with iodophor gauze. Computed tomography (CT) revealed a retroperitoneal abscess, concurrent with gas tracking along the fascial planes from the lateral abdominal wall to the perineum (Fig. 2D). Although we could not identify the exact cause of the retroperitoneal abscess at that moment, we believed it was the origin of this entire inflammatory process. Two consecutive microbiological cultures (aerobic plus anaerobic) of the tissue revealed the presence of extended-spectrum beta-lactamase (ESBL)-producing *Escherichia coli (E coli)*, which was susceptible to meropenem, piperacillin/tazobactam, amoxicillin/clavulanate potassium, amikacin, tetracycline, and gentamicin. On day 7, we maintained him on meropenem (1 g q.8 h) alone and applied continuous negative pressure wound therapy (NPWT) (V.A.C. Therapy, KCI, San Antonio, TX) at –125 mm Hg since the infection no longer appeared to be progressive. On days 10, 14, 18, and 22, the V.A.C. dressings were changed. Consecutive debridement demonstrated marked improvement with visible granulation tissue. On day 15, the antibiotics were changed to piperacillin/sulbactam (5 g q.8 h). On day 25, the wound was sutured, and a retroperitoneal drain was placed beside the sinus tract. On day 64, the patient was discharged, and a repeat CT scan revealed that the abscess was partially absorbed and the gas had disappeared (Fig. 3A). On day 81, the wound had healed completely. During the 32 months of follow-up, the patient recovered very well (Fig. 3B–D) and felt extremely satisfied.

## 3 Discussion

Retroperitoneal necrotizing fasciitis was first proposed by Rush et al^[4]^ in 1991, and the incidence and mortality were unclear, as the literature was limited to case reports and case series. According to the previous literature, the primary sources of retroperitoneal necrotizing fasciitis include perforated appendicitis,^[5]^ perforated cecal diverticulitis,^[6]^ necrotizing pancreatitis,^[7]^ and chronic pyelonephritis.^[8]^ In our case, the idiopathic retroperitoneal abscess was assumed to be the source. There was another possibility that the retroperitoneal abscess may have been the result of a more superficial presentation, such as FG. However, first, the patient had mild diarrhea and anorexia and had not suffered any trauma or surgery before visiting. The right flank symptoms began 9 days before the scrotal symptoms. Besides, none of the common etiologies of FG had been found, such as anorectal abscess or genitourinary injury. Second, the main site of necrosis was the retroperitoneum and intermuscular fascia rather than the scrotum or any other sites, the process that the infection spread from the retroperitoneum to the genital area was feasible anatomically. Third, most retroperitoneal abscesses result from a renal or gastrointestinal process, but in a small number of patients there is no identifiable source.^[9,10]^ Lastly, *E coli* was the pathogenic bacteria in our case, which is the most commonly isolated bacteria in intra-abdominal infections in China.^[11]^ This helped us to confirm the speculation that the infection was derived from the retroperitoneal abscess. Thus, the retroperitoneal NSTI was assumed to be induced by the idiopathic retroperitoneal abscess in our patient.

DM is the most frequent comorbidity in patients with necrotizing fasciitis, with the prevalence ranging between 40% and 60%.^[12,13]^ However, DM was not a risk factor for mortality.^[14]^

Imaging tests play an important role in cases of suspected retroperitoneal necrotizing fasciitis. CT scan is the first choice. The sensitivity and specificity of CT were reported to be 100% and 81%, respectively.^[15]^ Important findings on CT scanning include asymmetric fascial thickening and enhancement, muscular edema, fat stranding, fluid collection, and abscess formation.^[16]^ Additionally, CT scans could help to reveal the etiology of the infection. However, imaging tests must never delay emergency surgical treatment.^[17]^

The keys to managing retroperitoneal NSTI are surgical debridement, appropriate antibiotics, and systemic supportive therapy. First, surgical intervention is life-saving and must be performed as early as possible, since a delay in treatment beyond 12 hours in fulminant forms of necrotizing fasciitis could prove fatal.^[18]^ The mortality rate is 9 times greater when primary surgery is performed 24 hours after the onset of symptoms.^[13]^ Surgical debridement should be repeated during the next 24 hours or later, depending on the clinical course of the necrosis and vital functions.^[13]^ In terms of wound closure, NPWT is a revolutionary technique to improve local blood flow, induce macro-deformation, granulation, and angiogenesis, and reduce edema and bacterial colonization.^[19]^ All these advantages make NPWT suitable for the treatment of NSTI. With the help of NPWT, skin and soft tissue defects can be sutured directly instead of using a skin or flap graft in many cases. The dressing should be changed every 48 to 72 hours or no less than 3 times a week. However, because of the high cost of NPWT, which has not been covered by medical insurance in China, we had to prolong the dressing change interval to every 4 to 5 days or sometimes twice a week.

Second, broad-spectrum antibiotics should be administrated immediately to cover all possible organisms if necrotizing fasciitis is suspected.^[18]^ The most commonly isolated bacteria are reported to be *Streptococci*, followed by *Staphylococci,* and *Bacteroides*.^[20]^*E coli* is a rare pathogen causing necrotizing fasciitis (4.7%),^[20]^ and ESBL-producing *E coli* has rarely been reported in retroperitoneal NSTI cases. However, we cannot exclude polymicrobial infection because of prior exposure to antimicrobials. The therapeutic regimen can be adjusted after the bacteriologic diagnosis is established. The duration of antibiotic therapy requires a comprehensive consideration of systemic signs and symptoms, local signs of infection, and laboratory tests. The mean duration of antibiotic therapy is 4 to 6 weeks.^[18]^

Third, systemic supportive therapy is also important. Nutritional support, fluid and electrolyte balance, blood product transfusion, blood sugar control, and other supportive therapies should be performed. Some patients might have to be transferred to the intensive care unit.

This case reminds us that ESBL-producing *E coli* can cause retroperitoneal abscesses, which may induce NSTI. If diagnosis and treatment are delayed, the infection will spread rapidly and widely. Aggressive debridement and broad-spectrum antibiotics should be administrated immediately, and NPWT is an effective adjuvant therapy for wound closure.
